# Piccolo Device for Treating Patent Ductus Arteriosus Beyond Premature Newborn Patients, a Novel Experience in South America: A Retrospective Study

**DOI:** 10.1002/hsr2.71361

**Published:** 2025-10-09

**Authors:** Moeeza Fatima, Husnain Ahmad, Fatima Qasim

**Affiliations:** ^1^ Shaikh Khalifa Bin Zayed Al Nahyan Medical and Dental College Lahore Pakistan; ^2^ Ch Pervaiz Elahi Institute of Cardiology Multan Pakistan

Dear Editor,

I read with great interest the article by Aristizabal et al., which reports the use of the Amplatzer Piccolo Occluder in older pediatric patients for patent ductus arteriosus (PDA) closure, a commendable attempt to expand the device's use beyond its FDA‐approved indication in neonates over 700 g. The authors report a high procedural success rate of 96.5% in 29 patients, with no device embolization or acute procedural complications, contributing valuable regional data from South America [1]. However, several scientifically significant limitations merit consideration to contextualize the findings more critically.

First, the study omits essential hemodynamic data. Parameters such as pulmonary‐to‐systemic flow ratio (Qp:Qs), mean pulmonary artery pressures (mPAPs), and left atrial dimensions are not reported, which limits the ability to quantify the physiological significance of PDA and assess procedural benefit, particularly in the 34.5% of asymptomatic patients. In contrast, landmark PDA device studies have included these measures to define closure thresholds and assess efficacy [2, 3].

Another notable omission is the lack of procedural safety metrics. There is no mention of fluoroscopy time, radiation exposure, or contrast volume, despite the known cumulative risks of ionizing radiation in pediatric patients. The Turkish multicenter Piccolo study reported a mean fluoroscopy time of 6.2 min and used this benchmark to evaluate interventional safety [4]. Without these procedural details, it is not easy to gauge the comparative safety profile of the device in this older and more diverse cohort.

Moreover, the physiological appropriateness of applying a neonatal‐designed device to older children is insufficiently addressed. Ductal tissue in older children tends to be less compliant and more fibrotic, which can impair complete endothelialization and affect long‐term stability of the device [5]. The authors do not discuss this age‐dependent remodeling nor provide echocardiographic or histologic data to support equivalency in device–tissue interaction across the age spectrum. Furthermore, six patients had associated structural cardiac lesions (e.g., ASD or VSD). Yet, the study does not analyze how these defects may influence PDA hemodynamics, mask closure effects, or predispose to complications. Previous work has demonstrated that coexisting shunts can significantly alter left‐to‐right flow dynamics and procedural outcomes, warranting subgroup analysis and postprocedure ventricular function assessment [6].

In conclusion, while Aristizabal et al. contribute valuable clinical data on off‐label Piccolo use in a resource‐limited setting, the scientific generalizability of their findings is limited by the absence of comprehensive hemodynamic measurements, anatomical detail, procedural safety metrics, and long‐term follow‐up. Future studies would benefit from prospective multicenter designs, standardized inclusion of Qp:Qs and ductal geometry, and at least 12 months of echocardiographic monitoring to capture delayed complications such as LPA stenosis or aortic obstruction. Until such data are available, extending neonatal‐specific devices to older pediatric populations should be approached with caution and guided by meticulous anatomic and physiologic evaluation.

## Author Contributions

**Moeeza Fatima:** conceptualization, writing – original draft, writing – review and editing. **Husnain Ahmad:** investigation, conceptualization, funding acquisition, writing – original draft, writing – review and editing, project administration, resources. **Fatima Qasim:** conceptualization, investigation, funding acquisition, writing – original draft.

## Transparency Statement

The authors confirm this work is honest and transparent.

## Data Availability

The authors have nothing to report.
